# Synthesis of a 2,2'-Bipyridyl Functionalized Oligovinylene-Phenylene Using Heck and Horner-Wadsworth-Emmons Reactions and X-ray Crystal Structure of *E*-(4-(4-Bromostyryl)phenyl)(methyl)sulfane

**DOI:** 10.3390/molecules17055724

**Published:** 2012-05-14

**Authors:** Orsolya Karácsony, Jeffrey R. Deschamps, Scott A. Trammell, Rafaela Nita, D. Andrew Knight

**Affiliations:** 1 Chemistry Department, Florida Institute of Technology, 150 West University Boulevard, Melbourne, FL 32901, USA; Email: okaracsony2009@my.fit.edu (O.K.); rnita2010@my.fit.edu (R.N.); 2 Center for Bio/Molecular Science and Engineering, US Naval Research Laboratory, 4555 Overlook Avenue, SW, Washington, DC 20375, USA; Email: jeff.deschamps@nrl.navy.mil (J.R.D.); scott.trammell@nrl.navy.mil (S.A.T.)

**Keywords:** oligovinylphenylene, HWE reaction, Heck coupling

## Abstract

The synthesis of a new 2,2'-bipyridyl functionalized oligovinylenephenylene (**OVP-5**) containing a methyl protected thiol using Heck coupling and the Horner-Wadsworth-Emmons reaction and is described. A key step involving a diisopropylcarbodiimide promoted dehydration of a stable β-hydroxyphosphonate intermediate was identified. The structure of precursor *E*-(4-(4-bromostyryl)phenyl)(methyl)sulfane (**1**) was determined using X-ray crystallography.

## 1. Introduction

Oligovinylphenylenes (OVPs) are conjugated molecules that have demonstrated application in optoelectronics [1], molecular devices [2,3], photonics [4], liquid crystals [5] and organic dye sensitizers [6]. Trammell and co-workers recently described a new strategy for the formation of self-assembled monolayers of quinones derived from OVPs with thiol and 2,5-dimethoxylaryl head groups. Attachment of the thiol functionalized OVP to a gold surface allowed the study of formal redox potentials and reaction rates [7]. 

2,2'-Bipyridine ligands have been used extensively in homogeneous catalysis [8,9,10,11,12,13,14]. Martell and co-workers showed early on that 2,2'-bipyridine Cu(II) complexes are among the most efficient in hydrolyzing selected phosphorofluoridates, a class of compounds closely related to phosphate esters [15]. Since then, another group has reported comprehensively on the hydrolysis of other phosphate esters with such complexes [16]. Recent work by Chang’s group showed that immobilized copper(II)-bipyridine complexes were even more efficient at hydrolysis than the soluble complexes [17]. For example, a copper-containing polymer prepared by free radical polymerization of a copper(II)-vinylbipyridine complex with trimethylolpropane trimethacrylate (TRIM) showed significantly enhanced hydrolysis over the free complexes [17].

**Figure 1:**
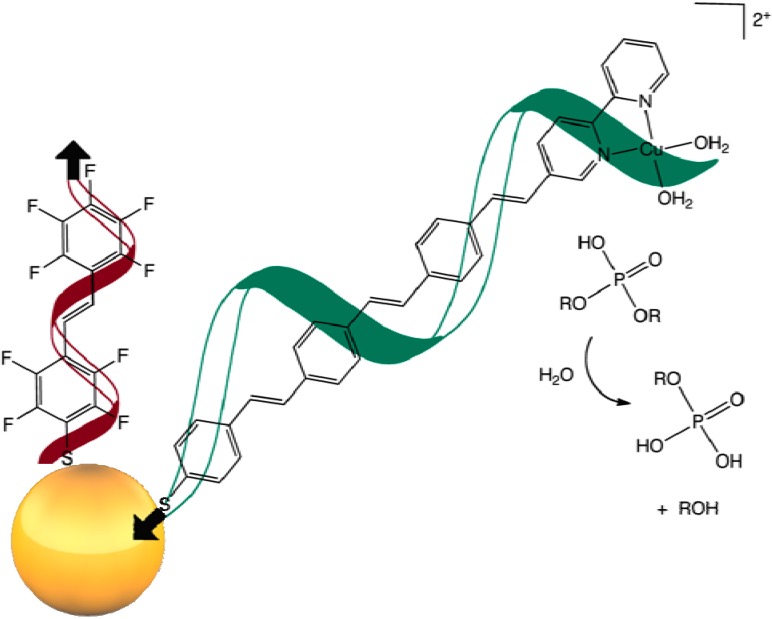
Electronic modulation of copper catalyzed organophosphate hydrolysis through an OVP-thiol bridge attached to a gold nanoparticle.

We recently initiated a program to investigate copper(II)-bipyridine functionalized molecular wires attached to gold nanoparticles with a view to the modulation of electronic and desolvation effects on catalytic phosphodiester hydrolysis (Figure 1). We propose that the electronic withdrawing properties of a highly fluorinated OVP ligand attached to a gold nanoparticle as shown, is likely to result in decreased electron density of a neighboring copper(II)-bipyridine functionalized molecular wire, in turn resulting in increased Lewis acidity of coordinated copper ion and consequent increase in catalytic activity.

Herein we report the synthesis and characterization of a 2,2'-bipyridine functionalized OVP ligand containing a protected thiol and the X-ray crystal structure of a synthetic precursor *E*-(4-(4-bromostyryl)phenyl)(methyl)sulfane.

## 2. Results and Discussion

**Scheme 1:**
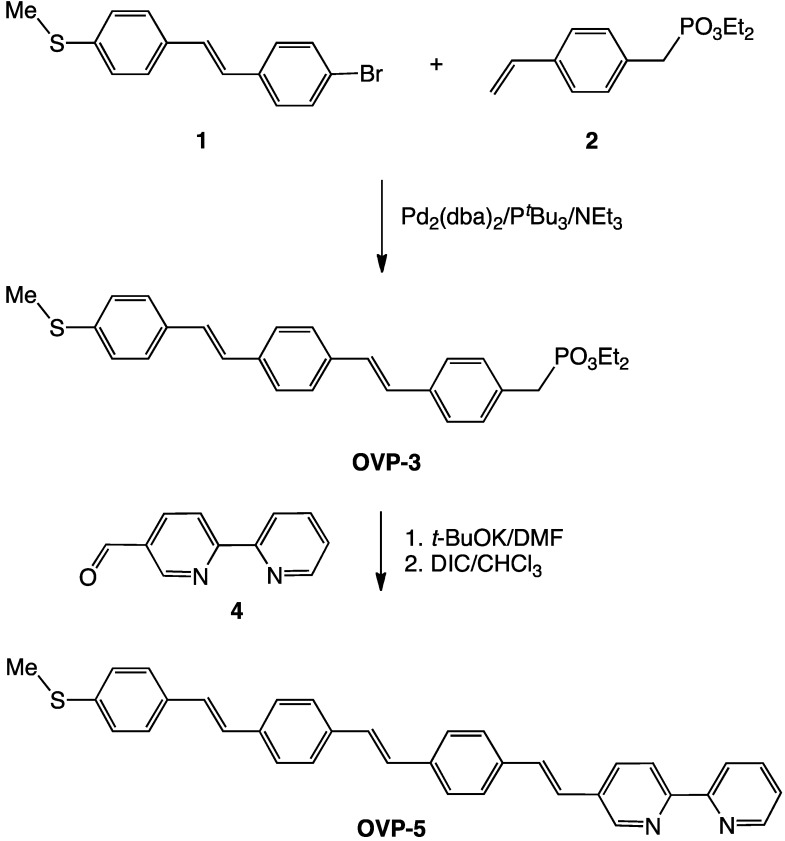
Synthesis of **OVP**-**5**.

Numerous strategies are available for the construction of OVPs. Our synthetic approach to a bipyridine functionalized molecular wire involved a sequential palladium catalyzed Heck coupling and a modified Horner-Wadsworth-Emmons reaction, as shown in Scheme 1. The arylbromide precursor **1** was synthesized, and the yield improved, using a modification of the literature procedure [6] via the reaction of diethyl 4-bromobenzylphosphonate with 4-(methylthiol)benzaldehyde which gave **1** as a spectroscopically pure amorphous solid. Crystallization of a portion of **1** from dichloromethane yielded colorless crystals which were of sufficient quality to be used directly for X-ray structure analysis.

**Figure 2:**
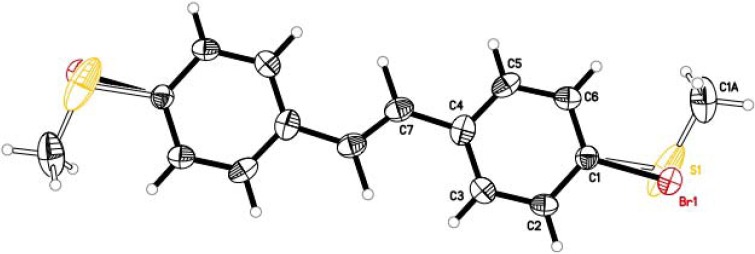
ORTEP style representation of **1** (displacement ellipsoids are shown at the 50% level).

The molecule crystallizes on a center of symmetry and therefore only half the molecule is present in the asymmetric unit. Because of this, the bromine atom and the CH_3_S-group are present at half-occupancy. The ORTEP representation of **1** is shown in Figure 2. X-ray analysis confirms the *E*-stereochemistry previously suggested from the ^1^H-NMR spectrum [6].

A palladium catalyzed Heck reaction with Pd_2_(dba)_3_·CHCl_3_/P*^t^*Bu_3_/NEt_3_ in dioxane was used to couple **1** with 4-vinylbenzylphosphonate (**2**) using conditions that are expected to give a high *E*/*Z* stereochemical ratio (Scheme 1) [18]. The OVP phosphonate **OVP-3** was isolated from the reaction mixture following silica gel flash column chromatography as a yellow solid in 70% yield and identified using ^1^H-NMR spectroscopy and mass spectrometry.

Only the *E*-isomer was observed based on the presence of the characteristic AB pattern with coupling constants ~16.0 Hz. Both compound **1** and 4-vinylbenzylphosphonate are recovered from the reaction mixture with no other species being identified. Modifications of the palladium catalyzed coupling reaction were then examined in an attempt to improve the yield of **OVP-3**, including a ligand-free Heck reaction with dimethylglycine as a promoter [19], the use of Pd(OAc)_2_ with Buchwald’s ligand, MePhos (2-(dicyclohexylphosphino)-2',4',6'-triisopropylbiphenyl) [20], and decreasing the mol% of palladium catalyst. In each case, no product **OVP-3** was observed.

**Scheme 2:**
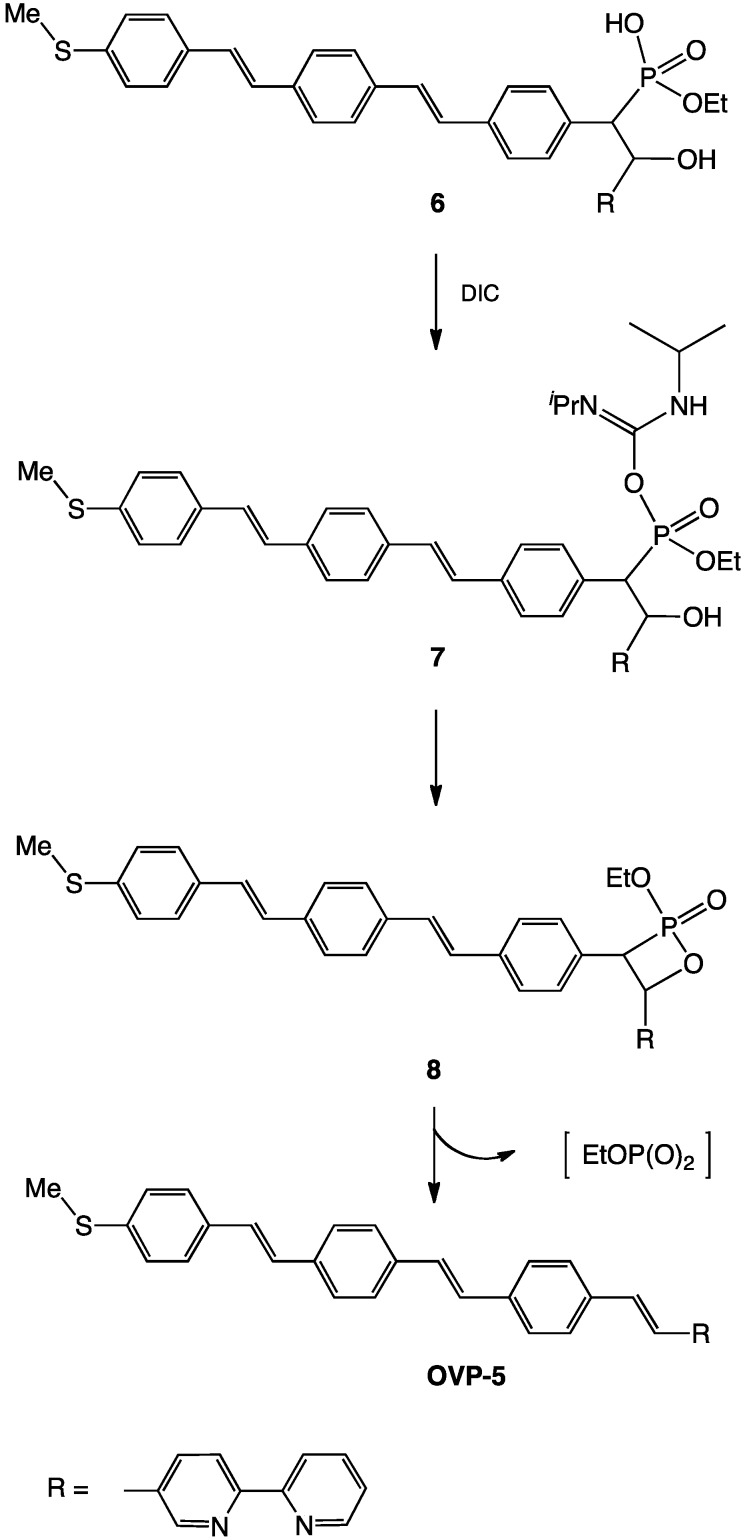
Formation of bipyridyl-functionalized **OVP-5** from β-hydroxyphosphonate **6**.

Reaction of the benzylphosphonate **OVP-3** with 2,2'-bipyridine-5-carbaldehyde **4** under Horner-Wadsworth-Emmons conditions with an excess of potassium *t*-butoxide followed by dehydration of the β-hydroxyphosphonate intermediate **6** (Scheme 1 and Scheme 2) with diisopropylcarbodiimide (DIC) gave **OVP-5**. Evidence for **6** was provided by in situ ^1^H-NMR spectroscopy and the intermediate was not further purified.

The phosphonic acid ethyl ester protons of **6** resonate at 1.41 and 4.01 ppm for the methyl and methylene protons respectively with associated coupling to the phosphorus nucleus. These values are typical for those previously reported for β-hydroxyphosphonic acid diethyl esters. We have tentatively assigned a multiplet and doublet (*J*_HP_ = 28 Hz) at 5.37 and 2.91 ppm to single protons alpha to the –OH and –PO_3_Et_2_ groups respectively. The formation of a stable, isolable β-hydroxyphosphonate intermediate in the HWE reaction is not entirely unexpected, as a carbanion-stabilizing group in the β-position is a requirement for elimination of phosphate from intermediate **8** and formation of an alkene. Compound **6** does not possess a withdrawing group in the β-position and cannot undergo elimination.

Reichwein and Pagenkopf recently reported the saponification of β-hydroxyphosphonic acid dimethyl esters followed by diisopropylcarbodiimide (DIC) dehydration to give the corresponding olefins [21]. We have discovered that the one-pot reaction of the phosphonic acid diethyl ester **OVP-3** with an excess of potassium *t*-butoxide directly followed by dehydration with DIC in CHCl_3_ resulted in conversion to the desired 2,2'-bipyridyl functionalized oligovinylphenylene **OVP-5** via intermediate **6** (Scheme 2). We tentatively suggest that excess *t*-butoxide anion is responsible for monodealkylation of the phosphonic acid ester **OVP-3** [2] to give **6** which then undergoes dehydration with the carbodiimide. Compound **OVP-5** was identified with ^1^H-NMR spectroscopy and ESI mass spectrometry. In situ ^1^H-NMR analysis of the DIC dehydration reaction shows complete disappearance of hydrogen atoms α to both hydroxy and phosphonate groups.

## 3. Experimental

### 3.1. General

All reactions were conducted under N_2_ using standard Schlenk line and catheter-tubing techniques unless otherwise stated. All ^1^H, ^31^P and ^13^C-NMR spectra were recorded on a Bruker 400 MHz NMR spectrometer and referenced to internal tetramethylsilane. ^31^P-NMR spectra were referenced to external H_3_PO_4_. Mass spectra were recorded on a Jeol AccuTOF JMS-T100LC mass spectrometer in ESI or DART mode, or were performed by HT Labs, San Diego, CA, USA. Solvents were obtained as follows and used without further purification: *t*-butanol, anhydrous dioxane, dimethylformamide (Sigma-Aldrich), acetone, methanol, dichloromethane (Fisher), and CDCl_3_, (Cambridge Isotope Laboratories). Silica gel (60–230 mesh, Sigma-Aldrich) and anhydrous MgSO_4_ (Fisher) were used as received. Flash chromatography was performed using silica gel (60–230 mesh, 60Å). TLC was performed on SiO_2_ plates with fluorescent indicator. Compounds were visualized under UV light or with phosphomolybdic acid stain. Reagents were obtained as follows and used without further purification: triethylamine, diisopropylcarbodiimide, 4-methylthiobenzaldehyde, 4-vinylbenzyl-phosphonate, Pd_2_(dba)·CHCl_3_ (Sigma-Aldrich), P*^t^*Bu_3_, potassium *t*-butoxide (Fisher). Diethyl (4-bromobenzyl)phosphonate was synthesized according to the literature procedure [6].

### 3.2. Synthesis of Compounds ***1**, **OVP-3*** and ***OVP-5***

*E-(4-(4-bromostyryl)phenyl)(methyl)sulfane* (**1**). A round bottom flask was charged with diethyl (4-bromobenzyl)phosphonate (10.0 g, 0.0325 mol) and a solution of *t*-BuOK (3.65 g, 0.0329 mol) in *t*-BuOH (40 mL) was slowly added with stirring. Then, a solution of 4-methylthiobenzaldehyde (2.10 g, 0.0362 mol) in *t*-BuOH (80 mL) was slowly added. The reaction solution was stirred for 1.5 h and then poured into water (525 mL). The resulting precipitate was collected via filtration, dissolved in acetone (115 mL) and the solution added to water (115 mL) to give a yellow precipitate which was collected via filtration. Crystallization from CH_2_Cl_2_ gave **1** as white crystals (7.05 g, 71%). ^1^H-NMR (CDCl_3_ δ: 7.46 (d, 2H), 7.41 (d, 2H), 7.35 (d, 2H), 7.24 (m, 2H), 7.01 (d, 2H), 2.50 (s, 3H). ^13^C-NMR (CDCl_3_ δ): 138.3 (s, SC_Ar_), 136.3 (s, C_Ar_), 133.8 (s, C_Ar_), 131.7 (s, C_Ar_), 128.7 (s, C_vinyl_), 126.9 (s, C_Ar_), 126.6 (s, C_Ar_), 121.2 (s, C_Ar_), 15.7 (s, SCH_3_). HRMS (DART) (*m/z*): 306.99 (100%), 304.99 (92%), 306.00 (11%), 307.99 (8%). Anal. Calcd for C_15_H_13_BrS: C, 59.02; H, 4.29. Found: C, 59.02; H, 4.31.

**OVP-3**. A 50 mL Schlenk flask was charged with 4-vinylbenzylphosphonate (0.163 g, 0.679 mmol), **1** (0.207 g, 0.679 mmol), triethylamine (0.290 mL, 2.03 mmol), Pd_2_(dba)·CHCl_3_ (0.006 g, 6 μmol), P*^t^*Bu_3_ (130 µL, 10 wt % in hexane) and anhydrous dioxane (20.0 mL). The mixture was refluxed for 20 h and the resulting solution allowed to cool to room temperature. Then, CH_2_Cl_2_ (100 mL) was added to the residue and the solution washed with brine (3 × 100 mL). The organic layer was dried over anhydrous MgSO_4_, the mixture filtered and solvent removed to give a yellow-green solid which was purified using column chromatography (SiO_2_, 10 g). A yellow band was eluted with 95:5 CHCl_3_:MeOH. Solvent was removed via rotary evaporation to give **OVP-3** as a yellow solid. Yield: 0.2284 g, 70%. ^1^H-NMR (CDCl_3_, δ): 7.47 (d, 2H, *J* = 8.0 Hz), 7.42 (d, 4H, *J* = 8.0 Hz), 7.36 (d, 4H, *J* = 8.0 Hz), 7.26 (d, 1H, *J* = 8.0 Hz), 7.24 (d, 1H, *J* = 8.0 Hz), 7.05 (d, 2H, *J* = 16.0 Hz), 6.98 (d, 2H, *J* = 16.0 Hz), 4.02 (m, 4H), 3.23 (d, 2H, *J* = 24.0 Hz), 2.51 (s, 3H), 1.25 (m, 6H). ^13^C-NMR (CDCl_3_, δ): 137.9 (s, C_Ar_), 136.7 (s, C_Ar_), 136.6 (s, C_Ar_), 136.0 (s, C_Ar_), 136.1 (s, C_Ar_), 134.3 (s, C_Ar_), 131.1 (s, C_Ar_), 131.0 (s, C_Ar_), 130.1 (s, C_Ar_), 130.2 (s, C_Ar_), 128.2 (s, C_vinylic_), 128.1 (s, C_vinylic_), 128.0 (s, C_vinylic_), 127.7 (s, C_vinylic_), 126.9 (s, C_Ar_), 126.8 (s, C_Ar_), 126.8 (s, C_Ar_), 126.7 (s, C_Ar_), 126.7 (s, C_Ar_), 126.6 (s, C_Ar_), 62.1 (d, ^2^*J*_CP_ = 6.67 Hz, OCH_2_), 33.7 (d, ^1^*J*_CP_ = 138.0, CH_2_P), 16.4 (d, ^3^*J*_CP_ = 5.94 Hz, CH_2_CH_3_), 15.8 (s, SCH_3_). ^31^P-NMR (CDCl_3_, δ): 26.7 (s). HRMS (DART) (*m/z*): 479.18 (100%), 480.18 (24%), 478.18 (22%), 481.19 (7%). ESI-MS (*m/z)*: [C_28_H_31_O_3_PS]^+^ 478.

**OVP-5**. A 50 mL Schlenk flask was charged with a solution of **4** (18.4 mg, 0.100 mmol) and **OVP-3** (23.9 mg, 0.0500 mmol) in DMF (5.5 mL). The reaction flask was placed in an ice bath, and a solution of *t*-BuOK (0.0283 g, 0.257 mmol) in *t*-BuOH (3 mL) was slowly added with stirring. The solutions was allowed to warm to room temperature and stirred for 19 h. Then, CH_2_Cl_2_ (20 mL) was added and the reaction mixture was washed with water (4 × 20 mL). The organic layer was dried over MgSO_4_ and the solvent was evaporated to give **6** which was identified by ^1^H-NMR. Chloroform (0.5 mL) was added followed by DIC (0.0114 g, 90.0 μmol). The mixture was stirred for 4 h, filtered on a short silica gel plug and the solvent removed by rotary evaporation to give **OVP-5** (47.8 mg, 100%). ^1^H-NMR (CDCl_3_, δ): 9.1–6.2 (aromatic/vinylic CH), 2.60 (s, 3H). ESI-MS *m/z*: M^+^ 508, [M+H]^+^ 509, [M−4H]^+^ 504.

### 3.3. X-ray Analysis of ***1***

Formula: C_15_H_13_BrS; *M*_r_ = 305.22; crystal color and shape: colorless hexagonal plate, crystal dimensions = 0.097 × 0.234 × 0.531 mm; crystal system: monoclinic; space group *C*2/*c* (no. 15); *a* = 30.840(3) Å, *b* = 7.1498(7) Å, *c* = 5.8545(6) Å; α = γ = 90°, β = 96.531°; *V* = 1282.5(2) Å^3^; *Z* = 4; radiation used: Mo Kα (λ = 0.71073); T = 100(2) K. For more details see cif file in the supplementary material. CCDC 881505 contains the supplementary crystallographic data for this paper. These data can be obtained free of charge via www.ccdc.cam.ac.uk/conts/retrieving.html/ (or from the CCDC, 12 Union Road, Cambridge CB2 1EZ, UK; Fax: +44-1223-336-033; Email: deposit@ccdc.cam.ac.uk).

## 4. Conclusions

In summary, a new 2,2'-bipyridine functionalized OVP ligand with a protected thiol has been synthesized using a combination of Horner-Wadsworth-Emmons and palladium catalyzed Heck coupling reactions. Coordination of the ligand to copper(II), and attachment to gold nanoparticles will be reported in due course.

## Supplementary Material

Supplementary data (X-ray analysis in cif format) associated with this article can be found at: http://www.mdpi.com/1420-3049/17/5/5724/s1.

## References

[1] Garner L.E., Park J., Dyar S.M., Chworos A., Sumner J.J., Bazan G.C. (2010). Modification of the optoelectronic properties of membranes via insertion of amphiphilic phenylene oligoelectrolytes. J. Am. Chem. Soc..

[2] Davis W.B., Svec W.A., Ratner M.A., Wasielewski M.R. (1998). Molecular wire behaviour in *p*-phenylenevinylene oligomers. Nature.

[3] Kushmerick J.G., Holt D.B., Pollack S.K., Ratner M.A., Yang J.C., Schull T.L., Naciri J., Moore M.H., Shashidar R. (2002). Effect of bond-length alternation in molecular wires. J. Am. Chem. Soc..

[4] Gao F., Xu Z.-Z., Yu Y.-H., Wang Q., Zhang H.-L., Fu H.-B. (2010). Strong two-photon excited fluorescence and stimulated emission from an organic single crystal of an oligo(phenylene vinylene). Angew. Chem..

[5] Goel M., Jayakannan M. (2010). Supramolecular liquid crystalline π-conjugates: The role of aromatic π-stacking and van der Waals forces on the molecular self-assembly of oligophenylenevinylenes. J. Phys. Chem. B.

[6] Im H., Kim S., Park C., Jang S.-H., Kim C.-J., Kim K., Park N.-G., Kim C. (2010). High performance organic photosensitizers for dye-sensitized solar cells. Chem. Commun..

[7] Trammell S.A., Moore M., Schull T.L., Lebedev N. (2009). Synthesis and electrochemistry of self-assembled monolayers containing quinone derivatives with varying electronic conjugation. J. Electroanal. Chem..

[8] Hassink M., Liu X., Fox J.M. (2011). Copper-catalyzed synthesis of 2,4-disubstituted allenoates from α-diazoesters. Org. Lett..

[9] Ye X., Xie C., Pan Y., Han L., Xie T. (2010). Copper-catalyzed synthesis of α-amino imides from tertiary amines: Ugi-type three-component assemblies involving direct functionalization of sp^3^ C-Hs adjacent to nitrogen atoms. Org. Lett..

[10] Boersma A.J., Klijn J.E., Feringer B.B., Roefles G. (2008). DNA-based asymmetric catalysis: Sequence-dependent rate acceleration and enantioselectivity. J. Am. Chem. Soc..

[11] Ricardo C., Matosziuk L.M., Evanseck J.D., Pintauer T. (2009). Strong coordination of tetraphenylborate anion to copper(I) bipyridine and phenanthroline-based complexes and its effect on catalytic activity in the cyclopropanation of styrene. Inorg. Chem..

[12] Csonka R., Kaizer J., Giorgi M., Réglier M., Hajba L., Mink J., Speier G. (2008). Oxidative C-H and C-C bond cleavage by a (2,2'-bipyridine)copper(I) chloride complex. Inorg. Chem..

[13] Saito T., Moore H.D., Hickner M.A. (2010). Synthesis of midblock-sulfonated triblock copolymers. Macromolecules.

[14] Niu J., Zhou H., Li Z., Xua J., Hu S. (2008). An efficient Ullmann-type C−O bond formation catalyzed by an air-stable copper(I)-bipyridyl complex. J. Org. Chem..

[15] Courtney R.C., Gustafson R.L., Westerback S.J., Hyytiainen H., Chaberek S.C.Jr., Martell A.E. (1957). Metal chelate compounds as catalysts in the hydrolysis of isopropyl methylphosphonofluoridate and diisopropylphosphorofluoridate. J. Am. Chem. Soc..

[16] Morrow J.R., Trogler W.C. (1988). Hydrolysis of phosphate diesters with copper(II) catalysts. Inorg. Chem..

[17] Hartshorn C.M., Singh A., Chang E.L. (2002). Metal-chelator polymers as organophosphate hydrolysis catalysts. J. Mater. Chem..

[18] Littke A., Fu G.C. (2001). A versatile catalyst for Heck reactions of aryl chlorides and aryl bromides under mild conditions. J. Am. Chem. Soc..

[19] Reetz M.T., Westermann E., Lohmer R., Lohmer G. (1998). A highly active phosphine-free catalyst system for Heck reactions of aryl bromides. Tetrahedron Lett..

[20] Billingsley K., Buchwald S.L. (2007). Highly efficient monophosphine-based catalyst for the palladium-catalyzed Suzuki-Miyaura reaction of heteroaryl halides and heteroaryl boronic acids and esters. J. Am. Chem. Soc..

[21] Reichwein J.F., Pagenkopf B.L. (2003). A new Horner-Wadsworth-Emmons type coupling reaction between nonstabilized β-hydroxy phosphonates and aldehydes or ketones. J. Am. Chem. Soc..

[22] Ho T.-L., Fieser M., Fieser L. (2006). Fieser and Fieser’s Reagents for Organic Synthesis.

